# Nutritional Biomarkers and Factors Correlated with Poor Sleep Status among Young Females: A Case-Control Study

**DOI:** 10.3390/nu14142898

**Published:** 2022-07-14

**Authors:** Sara AL-Musharaf, Lama AlAjllan, Ghadeer Aljuraiban, Munirah AlSuhaibani, Noura Alafif, Syed Danish Hussain

**Affiliations:** 1 Department of Community Health Sciences, College of Applied Medical Sciences, King Saud University, Riyadh 11451, Saudi Arabia; 442202852@student.ksu.edu.sa (L.A.); galjuraiban@ksu.edu.sa (G.A.); 441203145@student.ksu.edu.sa (M.A.); nalafeef@ksu.edu.sa (N.A.); 2 Riyadh Biochemistry Department, College of Science, King Saud University, Riyadh 11451, Saudi Arabia; danishhussain121@gmail.com

**Keywords:** PSQI, poor sleep, good sleep, sleep quality, sleep status, polyphenols, insulin resistant, HOMA-IR

## Abstract

Poor sleep status is associated with several health problems. Nutritional biomarkers and factors related to poor sleep are understudied. This study aimed to identify nutrition biomarkers and factors related to sleep status in healthy young Saudi females. The study included 92 normal-weight and obese Saudi females aged 19–25. Fasting blood glucose, insulin, and lipid profiles were measured. Insulin resistance was calculated on the basis of the homeostasis model assessment of insulin resistance (HOMA-IR) method. Anthropometric, stress, physical activity, and dietary data were collected. Data on the polyphenol content in foods were retrieved from the Phenol-Explorer database. The sleep status was assessed using the Pittsburgh sleep quality index (PSQI). Associations between variables were assessed using the multiple logistic regression model. Around 76% of the participants had poor sleep status (PSQI > 5). Multiple logistic regression reported high polyphenol intake as a protective factor against poor sleep (OR 0.24; 95% CI 0.07–0.83; *p* = 0.03) and HOMA-IR as an independent risk for poor sleep (OR 4.97; 95% CI 1.11–22.31; *p* = 0.04). Other nutritional biomarkers and factors, such as BMI, lipid profile, and vitamins, revealed a trend but were not significant. In conclusion, poor sleep status is associated with insulin resistance and low polyphenol intake among women of reproductive age.

## 1. Introduction

Good sleep status is crucial for an individual’s physiological, psychological, and cognitive well-being [1], especially among young adults [2]. Internationally, and in Saudi Arabia, studies have reported a high prevalence of poor sleep status (reported in 65% of adults in the United States [3] and 70% of college students in Saudi Arabia [4]), with a higher prevalence in females than in males [3,4]. Generally, poor sleep status is associated with several adverse health conditions, such as weight gain and obesity [5], diabetes mellitus [6], abnormal glucose metabolism [7], inflammation [8], and mortality [6]. Sleep disturbances may be particularly obvious in specific populations, such as females and the elderly [9]. Poor sleep status was also observed among undergraduate students and linked to the stress they face throughout their studies [10]. Since poor sleep status can adversely affect female reproductive health [11] and quality of life [12], further investigation is crucial in this specific group.

Various well-established risk factors, such as genetic factors [13], obesity [5], depression [6], and anxiety [14], contribute to poor sleep status. Nutritional factors, including energy and macronutrient intake, have recently garnered interest in research related to sleep status [15]. However, only a few studies have focused on other nutrients, such as vitamins, minerals, fibers, polyphenols, lipids, and lipoproteins, in relation to sleep status. Some vitamins deficiencies, such as the deficiency of vitamin B12 and vitamin D, have been associated with poor sleep status in young adults [16,17]. More recently, low serum high-density lipoprotein cholesterol (HDL-C), hyperglycemia, and insulin resistance have been independently linked to poor sleep status in adults and elderly people with diabetes or metabolic syndrome [6,18].

Another nutrient possibly associated with sleep is polyphenols [19]. Polyphenols are organic compounds found abundantly in plant-based foods and are classified into numerous groups, such as flavonoids, phenolic acids, and phytoestrogens [20]. A review study proposed that dietary polyphenols in fruits and vegetables are related to several potential health benefits, including optimal sleep [21]. Notably, only a few studies have investigated polyphenols in relation to better sleep quality, duration, and time to go to sleep among obese or normal-weight individuals [19,22,23]. Godos et al. [19] reported that a higher intake of certain polyphenols is associated with better sleep quality, specifically in normal-weight individuals [19].

Moreover, although obesity, diabetes, and hyperglycemia have been linked to poor sleep status [24,25,26], insulin resistance has been less investigated, especially among apparently healthy adults. There is an alarming increase in insulin resistance, as presented by impaired glucose tolerance, among young adults globally and in the Middle East and North Africa, affecting 10.6% and 11.2% of young adults, respectively [27]. Thus, another risk factor that may be related to poor sleep status among young adults is insulin resistance. So far, few studies have addressed insulin resistance along with poor sleep status among obese [28] and diabetic populations [29]. Hashemipour et al. [28] found that insulin resistance in overweight or obese individuals was associated with lower-quality sleep and, for each unit increase in the total sleep quality score, there was a 1.08 times elevated risk of insulin resistance [28].

Young females face multiple biological, behavioral, social, and environmental variables that put females of childbearing age at risk for sleep deprivation, which may contribute to short- and long-term health effects [11] and affect their academic performance [9]. Understanding how different nutrition biomarkers and factors, such as dietary intake, lipid profile, insulin, stress, and physical activity, are linked with poor sleep status is crucial, especially in young adults. To the best of our knowledge, no previous studies have assessed dietary polyphenols or insulin resistance in association with poor sleep status in young Arabian women. Therefore, the aim of this study is to explore different nutrition biomarkers and factors in association with poor sleep status among apparently healthy young Saudi females.

## 2. Materials and Methods

### 2.1. Study Design

A case-control study (obese vs. normal body weight) was conducted using female participants recruited from the King Saud University (KSU) campus between January 2019 and March 2020. Social media, the university’s Tawasul service, and flyers were used to recruit participants. Participants were 19–25 years old. After screening 400 participants, 290 participants were asked to book an appointment at the nutrition clinic of the Applied Medical Science College, KSU, to undergo a comprehensive assessment. A total of 92 participants were included in this study and divided on the basis of sleep status.

#### Sample Size

The sample size was calculated at a 5% significance level and an 80% power level. Therefore, a total of 92 participants were required, divided into two groups, normal weight and obese, 46 participants per group. The final estimated sample size was 99 participants, with a 10% dropout rate, comparable to previous findings. Post hoc power analysis for the difference in the mean polyphenol levels between the two groups found that this study had achieved a statistical power of more than 90%, with the effect size of 1.15 at 95% CI using *t*-test.

Those who were excluded were women who were (1) non-Saudi, (2) pregnant, (3) younger than 19 years or older than 25 years, (4) underweight (BMI < 18.5 kg/m^2^) or overweight (BMI > 24.9 to < 30 kg/m^2^), or (5) diagnosed with endocrine diseases, blood disorders, digestive system diseases (including acute or chronic diarrhea in the preceding 8 weeks), psychological disorders, oncological diseases, or metabolic disorders. This study is a part of a parent study that has been approved by the Research Ethics Committee of the Deanship of Scientific Research (KSU-IRB no. E-019-3625). All participants provided informed consent before participation.

### 2.2. Data Collection

On the day of the visit, the participants completed all comprehensive assessment phases, which were directed by a trained clinical dietitian, and this entire assessment took about 30–45 min. The comprehensive assessment entailed the following: (1) a blood sample was extracted, (2) the anthropometrics were measured, (3) a bioelectrical impedance analysis was performed, and (4) a face-to-face interview were conducted with the use of standardized and verified Arabic-validated questionnaires and taking date of birth (detailed below).

#### 2.2.1. Anthropometric Measurements

Anthropometrics were assessed using standardized procedures following the study protocol. The average of two repeated measures was used to increase the accuracy. Digital Pearson Scale was used to measure participants’ weights and heights (Digital Pearson Scale; ADAM Equipment Inc., Oxford, CT, USA). The BMI was calculated by dividing the weight in kilograms by the square of the height in meters (kg/m^2^) using the World Health Organization (WHO) BMI classification [30]. Waist circumference (WC) and hip circumference (HC) were measured with a non-stretchable tape using the WHO measurement protocol. If the variance between the reported measurements was higher than 2 cm, another measurement was taken. The WHR was computed by dividing the WC by the HC [31]. Body composition was analyzed by bioelectrical impedance analysis (770 Bioelectrical Impedance Analyzer; In-Body, Seoul, Korea), representing body fat mass, protein, and fluid percentage [32].

#### 2.2.2. Biochemical Blood Analysis

Blood samples were collected in the fasting state (≥10 h) from the participants and then transferred to the CBCD laboratory to analyze fasting blood glucose (FBG), insulin, lipid profile (HDL-C and total cholesterol (TC)), and TG levels. A biochemical analyzer was used to determine the lipid profile (Konelab, Espoo, Finland). Previously published formulas were used to compute the LDL-C [33]. A LIAISON XL analyzer was used to evaluate insulin levels (DiaSorin, Saluggia, Italy). The homeostatic model assessment of the insulin resistance (HOMA-IR) index was then computed [34]. HOMA-IR was categorized into two groups on the basis of a previous study [35], according to which HOMA-IR ≥ 1 means there is insulin resistance and HOMA-IR < 1 means there is no insulin resistance.

The electrochemiluminescence binding assay (ECLIA) was used to determine the serum levels of total 25(OH)D. Vitamin D were categorized as follows based on 25(OH)D concentrations: A severe deficiency was defined as <25 nmol/L; a non-severe deficiency was defined as ≥25 nmol/L [36]. Serum vitamin B12 levels were also assessed using an electrochemiluminescent immunoassay by a Roche Cobas e411 immunoassay analyzer (Roche Diagnostics, Munich, Germany).

#### 2.2.3. Sleep Index

The Arabic version of the Pittsburgh Sleep Quality Index (PSQI) was used to evaluate the participants’ sleep quality and disruptions in the month preceding the data collection date [37]. Subjective sleep quality, sleep latency, sleep length, sleep efficiency, sleep disruption, usage of sleep medicine, and daytime dysfunction were the seven components of the PSQI. Each component had four scores (not during the past month, less than once a week, once or twice a week, and three or more times a week). The higher total scores indicated a poorer sleep quality, as presented by a score > 5 [37].

#### 2.2.4. Stress Scale

The Arabic validated Perceived Stress Scale (PSS) was used to assess participants’ stress perceptions over the previous month [38]. This scale contained 10 components, each with five scores [38], including questions about stressful life events [38].

#### 2.2.5. Physical Activity (PA)

The Arabic version of the Global Physical Activity Questionnaire (GPAQ) was used to assess the participants’ physical activity [39]. The questionnaire assessed several components of physical activity, such as intensity, duration, and frequency [39].

#### 2.2.6. Dietary Data

A trained clinical dietitian collected all dietary data in a face-to-face interview, and both FFQ and 24-h dietary recall were used in this study. Using an FFQ developed by the Saudi Food and Drug Authority (SFDA), each participant was asked about her regular food consumption in the previous year [40]. The SFDA-FFQ was in Arabic and covered 133 food items with closed-ended questions. In addition, there were open-ended questions at the end of the questionnaire to collect information regarding unspecified food products [40]. This questionnaire provided us data on the intake of macro- and micronutrients, including vitamin D and vitamin B12.

To improve the accuracy of the dietary estimation derived from the SFDA-FFQ, 20% of the study participants were requested to complete a multiple-pass 24-h food recall. The degree of accuracy between the data provided in the 24 h and the FFQ ranged between r = 0.42 and r = 0.63 [41].

##### Polyphenol Intake

The Phenol-Explorer database (www.phenol-explorer.eu, accessed on 5 July 2021) was used to gather data on the polyphenol content of foods [42]. The database contains more than 400 foods, but the SFDA-FFQ contains 133 food items. Hence, unless otherwise stated in the next section, most of the foods in the SFDA-FFQ were available in the database. The amount of polyphenol in recipes and mixed foods was determined on the basis of the ingredients. The retention and yield factors of processed food were considered.

##### Estimation of Dietary Polyphenol Intake

The dietary polyphenol intake was estimated by calculating the polyphenol content reported in milligrams per 100 g or milliliter (mg/100 g or mg/100 mL) in the Phenol-Explorer database. The polyphenol content in the SFDA-FFQ Excel file is reported in milligrams per day. The Phenol-Explorer database contains five experimental methods for determining the polyphenol content in foods [43]. In this study, high-performance liquid chromatography (HPLC) was used to calculate the total polyphenol content [44]. Moreover, some extrapolations were carried out for missing data.

The total polyphenol content was computed by multiplying the sum of all individual polyphenols consumed by the quantity and frequency of food consumption. The total polyphenol content is presented in mg per day [45]. Finally, polyphenol intake is presented per 1000 kcal/day of total energy intake. On the basis of the median, dietary polyphenols consumption was divided into two categories: low intake and high intake (≤252 vs. >252 mg/1000 kcal/day). Additional categories were developed by calculating the median consumption in the case group (low intake ≤ 236 mg/1000 kcal/day vs. high intake > 236 mg/1000 kcal/day) and in the control group (low intake ≤ 281 mg/1000 kcal/day vs. high intake > 281 mg/1000 kcal/day).

### 2.3. Statistical Analysis

IBM SPSS statistics were used to evaluate the data sets (version 24; IBM software, Armonk, NY, USA). Quantitative data were checked for normality and skewness. Continuous variables were presented as means and standard deviations, whereas frequencies were provided as absolute numbers and percentages. To compare categorical variables, Pearson’s chi-square analysis was applied, and the independent sample *t*-test was used to compare polyphenol levels among participants.

The multiple logistic regression model was used to assess the association between poor sleep status as a dependent variable and different nutritional biomarkers and factors, including BMI, HDL, HOMA IR (≥1), energy intake, total grains, and high polyphenol (>median), as independent variables. Statistical significance was defined as a *p*-value < 0.05 and a confidence range of 95%.

## 3. Results

### 3.1. The General, Anthropometric Measurements, and Biochemical Analysis

The average age of the females was 21.1 (SD ± 1.5) years. In all, 76.1% of the participants had poor sleep status, with a PSQI score > 5, while 23.9% of the participants presented good sleep. The poor-sleep group had a higher BMI (good sleep 24.8 ± 6.0; poor sleep 29.7 ± 8.2 kg/m^2^; *p* = 0.004) and higher body fat (good sleep 39.3 ± 8.1; poor sleep 43.5 ± 9.6 kg; *p* = 0.39) than the good-sleep group. Other anthropometric parameters did not show statistical differences (Table 1). Furthermore, other sleep parameters did not show significance between people with normal weight and people with obesity (Supplementary Table S1).

Among clinical metabolic blood parameters, there were no significant differences between groups in terms of the lipid profile presented, with lower HDL cholesterol in the poor-sleep group compared with the good-sleep group (good sleep 1.1 ± 0.4; poor sleep 1.0 ± 0.3 mmol/L; *p* = 0.06) (Table 1). In terms of vitamin D deficiency, 36.5% of the poor-sleep group had a severe vitamin D deficiency (<25 nmol/L) compared with 10% of the good-sleep group, but the difference is not statistically significant after age and BMI adjustment (*p* = 0.07) (Table 1).

**Figure 1:**
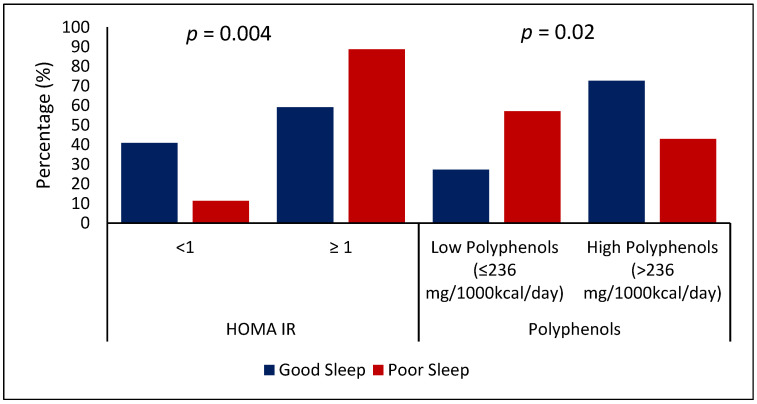
Association of HOMA-IR and polyphenols with sleep status. Note: Data are presented as absolute numbers and percentages, poor sleep as a dependent variable, and HOMA-IR (< or ≥1) and polyphenol (≤ or >median) as independent variables; *p* < 0.05 is considered significant; HOMA-IR: Homeostatic Model Assessment of Insulin Resistance.

The poor sleep group reported higher insulin levels (good sleep 6.0 μIU/mL; poor sleep 11.3 μIU/mL; *p* = 0.01) and higher HOMA-IR (good sleep 1.2; poor sleep 2.2; *p* = 0.03) after adjustment for age and BMI (Table 1). Similarly, when categorizing HOMA-IR into two groups (HOMA-IR ≥ 1), 88.7% of the poor-sleep group had HOMA-IR ≥ 1 in comparison to 11.3% with HOMA-IR < 1 and 59.1% of the good-sleep group had HOMA-IR ≥ 1 in comparison to 40.9% with HOMA-IR < 1 (*p*-value = 0.004) (Figure 1).

### 3.2. Dietary Intake and Lifestyle Characteristics

Furthermore, the study looked into some lifestyle parameters, including diet, physical activity, and stress, according to sleep status (Table 2). The poor-sleep group reported a higher energy intake in contrast to the good sleep group (good sleep 3049 kcal/day; poor sleep 3656 kcal/day; *p* = 0.07). In terms of macronutrients, for example, there was higher fat intake in the poor-sleep group (poor sleep 39.5% of total kcal; good sleep 37.1% of total kcal; *p* = 0.20).

According to the food group, in the poor sleep group, the total intake of fruits, grains, and polyphenols was statistically significant lower, after adjusting for age and BMI: fruit intake (good sleep 139 g/1000 kcal; poor sleep 94 g/1000 kcal; *p* = 0.02) and grain intake (good sleep 159 g/1000 kcal; poor sleep 134 g/1000 kcal; *p* = 0.04).

In addition, the good-sleep group reported a higher total intake of polyphenols (good sleep 349 mg/1000 kcal; poor sleep 237 mg/1000 kcal; *p* = 0.030) (Table 2). When categorizing polyphenol intake into two groups based on its median-to-low intake (≤236 mg/1000 kcal/day) vs. high intake (>236 mg/1000 kcal/day), 72.7% of the good-sleep group consumed more polyphenols than the poor-sleep group (*p*-value = 0.02) (Figure 1). Moreover, the sample was categorized into two groups based on serum HOMA-IR, where <1 represents a higher degree of insulin sensitivity and ≥1 represents insulin resistance. Around 88.7% of the poor-sleep group had higher insulin resistance (HOMA-IR < 1) compared to the good sleep group (11.3%) (*p*-value = 0.004) (Figure 1). In regard to physical activity and stress, there were no significant differences between the groups (Table 2).

### 3.3. The Association between BMI, HDL, HOMA-IR, Dietary Intake, and Sleep Status

Table 3 presents the odds of poor sleep status against selected parameters that showed significance in Table 1 and Table 2. Crude odds ratios of BMI and HOMA-IR with significant *p*-values indicate that higher BMI and HOMA-IR are associated with poor sleep. Furthermore, a significant crude odds ratio of high polyphenol shows that a high polyphenol intake is associated with good sleep. Multiple logistic regression identified HOMA-IR as an independent risk factor and high polyphenol as a protective factor against poor sleep, with Nagelkerke R Square of 31.3%.

## 4. Discussion

To the best of our knowledge, this is the first study to explore different nutritional biomarkers and factors in association with poor sleep status among young Saudi women. Seventy-six of the participants suffered from poor sleep. This study identified HOMA-IR as an independent risk for and high polyphenol as protective factor against poor sleep. Other nutritional biomarkers and factors showed a trend that failed to reach significance in the regression, i.e., BMI, lipid profile, vitamins (D and B12), and total energy intake.

### 4.1. Insulin Resistance and Sleep

Observational and experimental studies on the relationship between insulin resistance and sleep status are limited [28,46,47,48], with only a few observational studies [28,46,48]. Our study results extend the findings of scarce previous research [28,46,47,48], indicating that HOMA-IR is independently related to a five-fold higher risk of poor sleep. In addition, participants who had poor sleep showed higher insulin levels compared with those with good sleep (poor sleep 11.3 μIU/mL; good sleep 6.0 μIU/mL; *p* = 0.01). 

Similar to our results, a recent cross-sectional study of 612 adults (≥20 years) with BMI ≥ 25 kg/m^2^ either with or without insulin resistance showed that the total score of sleep quality (using PSQI) was significantly lower in participants who had HOMA-IR > 3.4 compared with those who did not have insulin resistance [28]. A prospective case-control study that measured sleep objectively using the actigraph device found that glycemic status influences sleep quality, indicating a significant difference in sleep efficiency between people with type 2 diabetes mellitus, prediabetes, and normal glucose tolerance (86%, 88%, and 90%, respectively; *p* = 0.04) [46]. A randomized controlled trial involving 16 apparently healthy men revealed a link between partial sleep deprivation (<4.25 h of sleep) and significantly higher peripheral insulin resistance [46]. In contrast, Joo et al. found a significant association between sleep duration and impaired fasting glucose in men but not in women [47]. The contradictory results could be due to different tools measuring sleep, sleep duration vs. sleep quality, different study designs, and the varied nature of the sample.

Our findings indicate that poor sleep status is correlated with changes in appetite-regulating hormones, increased sympathetic tone, and increased cortisol secretion, all of which can contribute to insulin resistance [6]. In addition, the relationship between sleep status and insulin resistance may be bidirectional. A prospective study over a six-year period found that the HOMA-IR score was a predictor of the incidence of obstructive sleep apnea [48].

The relationship between obesity and poor sleep has been well documented in the literature [49,50]. Poor sleep status has been associated with obesity and excess body fat among adults [50] and college students [49]. Our study showed a significantly higher BMI in the poor-sleep group compared to the good-sleep group (poor sleep 29.7 kg/m^2^; good sleep 24.8 kg/m^2^; *p* = 0.004), but the level was not significant. This can be explained by the nature of the sample, the study design, and the sleep measurement tool. Furthermore, an abnormal lipid profile has recently been associated with poor sleep [51]. Our study showed that a higher HDL level may protect against poor sleep by 80%, but the finding failed to reach significance (*p* = 0.06). The association between poor sleep and the HDL level was proven in a previous RCT, which showed that improvement in sleep quantity and quality is independently associated with a higher HDL level (β = 0.04; *p* = 0.03) [51].

### 4.2. Polyphenols and Sleep

Experimental and observational studies on the relationship between dietary polyphenols and sleep are scarce [19,22,23,52,53,54]. So far, only three observational [19,22,54] and two experimental studies [23,52] have been found to relate polyphenol intake with sleep quality. This study proposed that participants who consume higher amounts of polyphenols are independently protected by 76% from poor sleep.

Similar to our study, a recent Italian observational study presented that higher consumption of certain dietary polyphenols (some subclasses of flavonoid, phenolic acids, and lignans) could enhance the sleep quality in healthy adults with normal body weight, but the association has not been confirmed among overweight/obese participants [19]. An RCT investigated the effect of 30-day polyphenol supplementation on sleep quality in participants with subclinical sleep disturbances [23]. In the study, polyphenol supplementation significantly improved sleep quality (β = 0.11; CI 95% 0.01, 0.22; *p* = 0.008) and decreased the insomnia severity index (β = −0.1; CI 95% −2.0, −0.03; *p* = 0.044) compared to the placebo group [23]. This is in accordance with other RCT studies among apparently healthy adults with a BMI of 25–35 kg/m^2^, which found that sleep quality significantly improved by 43% in participants who receive 1000 mg/day of HolisFiit^®^ (a polyphenol-rich food supplement) for 16 weeks (*p* = 0.0001) [52].

However, one study did not find any association between sleep and polyphenols [53], and another showed a contradictory result in terms of sleep duration [22]. Noorwali et al. [22] conducted a cohort prospective study involving 13,958 females, with follow-ups over 4 years; it showed that a higher polyphenol intake was inversely correlated with sleep duration, i.e., a higher polyphenol intake led to shorter sleep [22]. However, Noorwali et al. did not use a validated sleep measurement tool [22]. Furthermore, these contradictory results could be justified by the high variability in polyphenol contents between foods, measurement of one type of polyphenol (resveratrol) [53], recall bias that may occur while collecting polyphenol information, and use of different tools to measure sleep along with varied study designs and participants.

The relation between sleep and polyphenols could be explained by a couple of points. Overall, healthy diets have a critical impact on sleep and the health implications connected with it and following a healthy plant-based diet is associated with good sleep quality. Pourreza et al. [54] conducted a cross-sectional study in 390 overweight and obese women aged 18–48 years old. They found that higher adherence to an unhealthy plant-based diet was significantly associated with a higher PSQI score (poor sleep quality) (β= 0.20; *p* = 0.04) [54]. According to St-Onge, plant-based diets enhance mitochondrial function, energy metabolism, and body composition, which may substantially improve sleep quality [55]. A plant-based diet is rich in fruits and vegetables, the major source of polyphenols in the diet [56]. In addition, the suggested mechanism behind the positive impact of polyphenols on the sleep status is the antioxidant effect of polyphenols, which may improve sleep quality via anti-inflammatory and antioxidant mechanisms [57,58].

Furthermore, emerging studies suggest that some individual dietary polyphenols, such as rosmarinic acid (phenolic acids) and epigallocatechin-gallate (flavonoids), may benefit sleep [59]. In the brain, rosmarinic acid displays significant antioxidant properties that provide neuroprotective benefits, which may influence sleep by modifying gamma-aminobutyric acid and acetylcholine [60]. Gamma-aminobutyric acid is the main inhibitory neurotransmitter in the human brain [61], but acetylcholine is the most important one [62,63]. Epigallocatechin-gallate may reduce secretion of the stress hormone (corticosterone) to downregulate the hypothalamic–pituitary–adrenal axis, which in turn exerts antianxiety and soporific effects [64,65]. Therefore, dietary polyphenols may play a role in enhancing sleep status. Nevertheless, more controlled trials on humans are needed to confirm the relationship between dietary polyphenols and sleep quality/duration.

### 4.3. Strength and Limitations

According to our research, the present study is the first in the Middle East to report a significant link between high polyphenol consumption and good sleep and a significant relationship between poor sleep and insulin resistance among young Saudi women. This study also used the Phenol-Explorer database, which is validated and widespread for estimating the polyphenol content of foods.

Our study has some limitations. First, it was part of a case-control study design, which implies that causation cannot be inferred by such an observational research study. Second, all approaches used for evaluating food consumption and dietary polyphenol intake provided only estimations, which can be affected by recall bias. However, the study to improve accuracy in evaluating dietary consumption combined SFDA-FFQ and multiple-pass 24-h food recall and showed a strong correlation. Third, since the PSQI for sleep assessment and the other questionnaires (PSS, and GPAQ) relied on subjective reporting, there was a probability of recall bias during data collection, though these questionnaires have been commonly used in recent scientific literature, representing the standard for scientific research. Finally, the study was conducted on young females only, so the results cannot be generalized to the general Saudi population.

## 5. Conclusions

In our study, 76.1% of the participants suffered from poor sleep. Insulin resistance emerged as a risk factor for poor sleep, whereas high polyphenol intake emerged as a protector. Thus, taking into consideration nutritional biomarkers can help in preventing poor sleep and its health complications [11]. Further studies with cohort and randomized control trials are needed to determine the relationship between nutritional biomarkers and sleep status.

## Figures and Tables

**Table 1:** Anthropometric and clinical-metabolic blood parameters according to PSQI sleep status.

Parameters	PSQI Classification		*p*-Value	*p*-Value *
	Good Sleep 22 (23.9%)	Poor Sleep 70 (76.1%)		
**Anthropometric Measurements**				
Age (years)	20.6 ± 1.0	21.2 ± 1.6	0.07	
BMI (kg/m^2^)	24.8 ± 6.0	29.7 ± 8.2	0.004	
WHR (ratio)	0.7 ± 0.1	0.7 ± 0.1	0.11	0.27
Fat (kg)	39.3 ± 8.1	43.5 ± 9.6	0.07	0.39
Protein (kg)	7.2 ± 1.0	7.8 ± 1.2	0.04	0.94
Fluid%	44.7 ± 5.8	41.3 ± 6.9	0.05	0.46
**Clinical-Metabolic Blood Parameters**				
Total cholesterol (mmol/L)	4.2 ± 1.4	4.0 ± 1.5	0.67	0.23
FBG (mmol/L)	4.4 ± 0.7	4.7 ± 0.7	0.10	0.40
HDL cholesterol (mmol/L)	1.1 ± 0.4	1.0 ± 0.3	0.15	0.06
LDL cholesterol (mmol/L)	2.9 ± 1.2	2.9 ± 1.4	1.00	0.49
Total cholesterol/HDL ratio	4.0 ± 1.6	4.3 ± 1.7	0.43	0.73
Triglyceride (mmol/L) #	0.7 (0.5–0.9)	0.8 (0.5–1.0)	0.29	0.68
Insulin (μIU/mL) #	6.0 (4.3–12.4)	11.3 (7.0–17.8)	<0.001	0.01
HOMA-IR #	1.2 (0.8–2.2)	2.2 (1.2–3.9)	<0.001	0.03
HOMA-β #	98.8 (64.1–274.4)	182.7 (131.3–306.1)	0.06	0.29
Vitamin D (nmol/L)	33.0 (26.4–41.4)	32.3 (22.8–46.8)	0.75	0.73
Severe vitamin D deficiency (<25 nmol/L)	2 (10.0)	23 (36.5)	0.02	0.07
Vitamin B12 (pg/mL)	477.3 (322.2–683.3)	389.6 (311.0–625.2)	0.46	0.60

Note: Data are presented as the mean ± the SD and the median (Quartile 1–Quartile 3) for normal and non-normal variables (#), respectively; *p* < 0.05 is considered significant; * indicates *p*-values adjusted for age and BMI. BMI: body mass index; WHR: waist-to-hip ratio; FBG: fasting blood glucose; HDL: high-density lipoprotein; LDL: low-density lipoprotein; HOMA-IR: Homeostatic Model Assessment of Insulin Resistance; HOMA-β: homeostasis model assessment of β-cell function.

**Table 2:** Dietary intake, physical activity, and stress according to PSQI sleep status.

Parameters	PSQI Classification		*p*-Value	*p*-Value *
	Good Sleep 22 (23.9%)	Poor Sleep 70 (76.1%)		
**Dietary Parameters**				
**Energy (kcal/day)**	3048.5 (2574.5–3539.7)	3656.3 (2700.9–4649.3)	0.08	0.07
Fat (% of total kcal)	37.1 (30.6–41.6)	39.5 (33.6–46.5)	0.11	0.20
Protein (% of total kcal)	14.1 (12.9–16.1)	13.4 (11.6–15.1)	0.15	0.16
CHO (% of total kcal)	48.6 (45.1–53.1)	45.0 (39.6–52.8)	0.18	0.33
Fiber (g/1000 kcal)	9.5 (8.0–12.6)	9.8 (7.9–11.4)	0.63	0.75
Na (mg/1000 kcal)	1459.9 (1278.0–1881.2)	1323.7 (1110.7–1637.9)	0.08	0.10
**Clinical-Metabolic Blood Parameters**				
K (mg/1000 kcal)	2070.0 (1418.2–2774.0)	1761.9 (1449.3–2680.0)	0.29	0.33
Ca (mg/1000 kcal)	39.2 (12.8–76.6)	54.2 (19.3–108.8)	0.33	0.86
P (mg/1000 kcal)	653.2 (527.0–1020.9)	597.2 (520.2–749.9)	0.25	0.27
Fe (mg/1000 kcal)	15.1 (9.9–20.8)	11.6 (9.2–17.6)	0.20	0.26
Vitamin B12 intake (mcg/day)	7.3 (5.8–12.3)	7.8 (5.0–13.8)	0.96	0.79
Vitamin D intake (IU/day)	3.3 (2.8–5.3)	4.1 (2.2–6.2)	0.71	0.82
Polyphenols (mg/1000 kcal)	348.6 (279.9–402.9)	237.2 (180.7–311.2)	0.004	0.03
Low Polyphenols (≤236 mg/1000 kcal/day)	194.8 (122.0–253.6)	188.2 (148.6–217.8)	0.69	0.75
High Polyphenols (>236 mg/1000 kcal/day)	392.2 (338.8–439.7)	330.6 (284.4–480.2)	0.13	0.88
**Food Groups**				
Total dairy (g/1000 kcal)	74.4 (43.5–96.9)	99.1 (51.9–132.8)	0.23	0.78
Total fruit (g/1000 kcal)	139.2 (78.1–316.9)	94.1 (55.2–164.9)	0.01	0.02
Total vegetables (g/1000 kcal)	201.4 (147.1–286.2)	175.0 (116.4–247.6)	0.18	0.30
Total grains (g/1000 kcal)	159.0 (128.0–194.6)	134.2 (87.7–174.9)	0.07	0.04
Total red meat	16.3 (14.6–19.4)	13.5 (7.7–18.6)	0.02	0.14
Total white meat	33.9 (29.0–39.8)	30.3 (17.4–40.7)	0.25	0.20
Total dairy (g/1000 kcal)	74.4 (43.5–96.9)	99.1 (51.9–132.8)	0.23	0.78
**Physical Activity Parameter**				
GPAQ score	8.1 ± 1.0	8.2 ± 1.1	0.86	0.79
**Stress Parameter**				
PSS score	16.9 ± 7.3	19.5 ± 6.0	0.09	0.11

Note: Data are presented as the mean ± the SD and the median (Quartile 1–Quartile 3) for normal and non-normal variables, respectively; *p* < 0.05 is considered significant; * indicates *p*-values adjusted for age and BMI. GPAQ: Global Physical Activity Questionnaire; PSS: perceived stress scale.

**Table 3:** Association between different nutritional biomarkers or factors and poor sleep status.

	Crude		Multiple Regression	
	OR (95% CI)	*p*-Value	OR (95% CI)	*p*-Value
BMI	1.10 (1.02–1.18)	0.02	1.09 (0.99–1.20)	0.08
HDL	0.32 (0.07–1.54)	0.16	0.19 (0.03–1.10)	0.06
HOMA-IR (≥1)	5.44 (1.71–17.32)	0.004	4.97 (1.11–22.31)	0.04
Energy intake (>2200 kcal)	0.95 (0.24–3.80)	0.94	2.37 (0.35–16.01)	0.38
High polyphenol (>median)	0.28 (0.10–0.80)	0.02	0.24 (0.07–0.83)	0.03
Total grains (>Median g/1000 kcal)	0.61 (0.23–1.63)	0.33	0.72 (0.22–2.30)	0.58
Nagelkerke R Square (*p*-value)	--	--	31.3%	0.003

Note: Data are presented as OR (95% CI); the multivariate logistic regression model includes poor sleep as a dependent variable and BMI, HDL, HOMA-IR (≥1), energy intake (>2200 Kcal), total grains, and high polyphenols (>median) as independent variables; *p* < 0.05 is considered significant. HOMA-IR: Homeostatic Model Assessment of Insulin Resistance; BMI: body mass index; HDL: high-density lipoprotein.

## Data Availability

Not applicable.

## Supplementary Materials

The following supporting information can be downloaded at: https://www.mdpi.com/article/10.3390/nu14142898/s1, Table S1: Sleep indices across normal weight and obese group.
