# Supplementary material for: Nutritional Biomarkers and Factors Correlated with Poor Sleep Status among Young Females: A Case-Control Study
Source: Nutrients. 2022 Jul 14;14(14):2898. doi: 10.3390/nu14142898 (PMC9320813; doi:10.3390/nu14142898)
Supplement: Supplementary file 1 [file nutrients-14-02898-s001.zip › nutrients-1802388-supplementary.pdf]

Supplementary Table 1. Sleep indices across normal weight and obese group [1,2]

Characteristics	Total (n=92)	Normal weight BMI = 18.5-24.9 (n=48)	Obese group BMI ≥30 (n=44)	p-Value
Total hours of sleep	5.0 ± 1.9	5.0 ± 1.8	4.9 ± 2.1	0.733
**Sleep components**				
**Quality**				
Very good	26 (28.3)	16 (33.3)	10 (22.7)	0.294
Fairly good	45 (48.9)	23 (47.9)	22 (50)	
Fairly bad	17 (18.5)	6 (12.5)	11 (25)	
Very bad	4 (4.3)	3 (6.2)	1 (2.3)	
**Latency**				
0	18 (19.6)	10 (20.8)	8 (18.2)	0.702
1 – 2	32 (34.8)	18 (37.5)	14 (31.8)	
3 – 4	26 (28.3)	11 (22.9)	15 (34.1)	
5 – 6	16 (17.4)	9 (18.8)	7 (15.9)	
**Duration**				
> 7 hours	16 (17.4)	7 (14.6)	9 (20.5)	0.433
6 – 7 hours	18 (19.6)	11 (22.9)	7 (15.9)	
5 – 6 hours	30 (32.6)	18 (37.5)	12 (27.3)	
< 5 hours	28 (30.4)	12 (25)	16 (36.4)	
**Efficiency**				
> 85%	67 (72.8)	36 (75)	31 (70.5)	0.594
75 – 84%	13 (14.1)	5 (10.4)	8 (18.2)	
65 – 74%	4 (4.3)	3 (6.2)	1 (2.3)	
< 65%	8 (8.7)	4 (8.3)	4 (9.1)	
**Disturbance**				
0	6 (6.5)	3 (6.2)	3 (6.8)	0.293
1 – 9	72 (78.3)	35 (72.9)	37 (84.1)	
10 – 18	14 (15.2)	10 (20.8)	4 (9.1)	
**Medication**				
Not during past month	87 (94.6)	44 (91.7)	43 (97.7)	0.241
Less than once a week	2 (2.2)	1 (2.1)	1 (2.3)	
Once or twice a week	3 (3.3)	3 (6.2)	0 (0)	
**Day Dysfunction**				
0	7 (7.6)	5 (10.4)	2 (4.5)	0.628
1 – 2	31(33.7)	17 (35.4)	14 (31.8)	
3 – 4	31 (33.7)	14 (29.2)	17 (38.6)	
5 – 6	23 (25)	12 (25)	11 (25)	
**PSQI Score**				
Good	14 (15.2)	8 (16.7)	6 (13.6)	0.686
Poor	78 (84.4)	40 (83.3)	38 (86.4)	

[1]Continuous variables are presented as (mean ± standard deviation (SD)); Median (1st quartile – 3rd Quartile) for non-normal continuous variables.
[2] Categorical and binary variables are presented as [n, (%)].
[3] The Pittsburgh Sleep Quality Index (PSQI)
